# Controlling the Bidirectional Circular Polarization States Using Ultrathin Back-to-Back Quarter-Wave Plates Cavity

**DOI:** 10.1038/s41598-017-15514-2

**Published:** 2017-11-10

**Authors:** Le Chang, Yue Li, Yongmin Liu

**Affiliations:** 1 Department of Electronic Engineering, Tsinghua University, Beijing, 100084 China; 2 Department of Mechanical and Industrial Engineering, Department of Electrical and Computer Engineering, Northeastern University, Boston, Massachusetts, 02115 USA

## Abstract

Efficiently manipulating the polarization states of electromagnetic waves is of great importance for communication, imaging, and sensing. In this paper, we aim to control the circular polarization states, e.g., left-hand, or right-hand, for the bidirectional radiated waves utilizing a pair of back-to-back quarter-wave plates, which are integrated within an ultrathin cavity. As an example, a bidirectional circularly polarized wave with the same helicity in forward and backward is generated based on numerical analyses, and proved by experiments in microwave region. The proposed ultrathin back-to-back quarter-wave plates cavity can be adopted to higher frequencies, e.g., terahertz and mid-infrared ranges, with lower metallic conductivity. The proposed method exhibits the advantages of compact dimension and low-cost implementation in engineering the bidirectional polarization states of electromagnetic waves.

## Introduction

Efficiently controlling the polarization states is essential not only in optics and photonics, but also in microwave region^1^. Circularly polarized (CP) waves, that is, right-hand circularly polarized (RCP) or left-hand circularly polarized (LCP) waves, are of importance in a communication system since the rotation effect for linear polarizations (e.g., Faraday rotation) can be eliminated^2^. Quarter-wave plates (QWPs) are usually used to convert an input linearly polarized (LP) wave to an output CP wave by introducing 90-degree phase difference between the two orthogonal LP components with equal magnitude. QWP, as an effective method to control the spin state of electromagnetic wave, can be achieved using birefringent materials in a bulky volume^3–5^ or using flatland structures based on metasurfaces^6–8^.

Here, we aim to control the polarization states of the bidirectional CP wave, which are with significant usage in the wireless sensing networks, portable access points, and relay systems, improving the system capacity and the stability of the communication link^9,10^. For this quest, there are various strategies using layered structures to generate the bidirectional CP wave with identical or opposite helicities. For example, slot apertures are a well-known method to produce bidirectional CP wave, but with intrinsically opposite helicities^11,12^. To achieve the identical helicities, structures with multiple layers (more than two layers) are indispensable, for example, a three-layered frequency selective surfaces or back-to-back patches with a common ground (three metallic layers and two dielectric layers)^13,14^. As an alternative method but with a bulky volume, spatial difference is utilized to achieve quarter-wavelength phase difference based on waveguide structures^15^. As a summary to the literature study, so far there have been no demonstrations of generating bidirectional CP waves with identical helicity using a single dielectric layer. Here, for the first time, a single and ultrathin dielectric layer structure with back-to-back QWPs (e.g., as an ultrathin cavity) is proposed to control the bidirectional CP wave with any helicities, including the important quest of identical CP state for forward and backward waves.

Two aspects can illustrate the engineering values of this work: (i) Why do we need bidirectional CP waves with identical helicity? (ii) Why must we use single dielectric layer? For the first question, an important application using the proposed structure is the low-cost and temporal wireless relay systems in narrow channel environments, for example, coal mine and tunnel channels^9,10^. Many engineered structures with bidirectional CP wave are required to identify the forward or backward waves. In contrast, for a structure with opposite CP states in forward and backward directions, the installment must be very careful to avoid mismatch of the CP states. One mistake of matching, for example, a receiving/transmitting RCP QWP is faced with a transmitting/receiving LCP QWP, will destroy the whole relay link for this temporal communication stations. Consequently, bidirectional wave with the same helicity along the forward and backward directions is highly desired^9,10,16,17^. For the second question, the proposed structure can be easily fabricated using the standard printed circuit board (PCB) process. As we all know, a multiple layered PCB is much more expensive than a single layered PCB. For the cost consideration, single layered structure is more preferred for mass production from engineering point of view.

**Figure 1:**
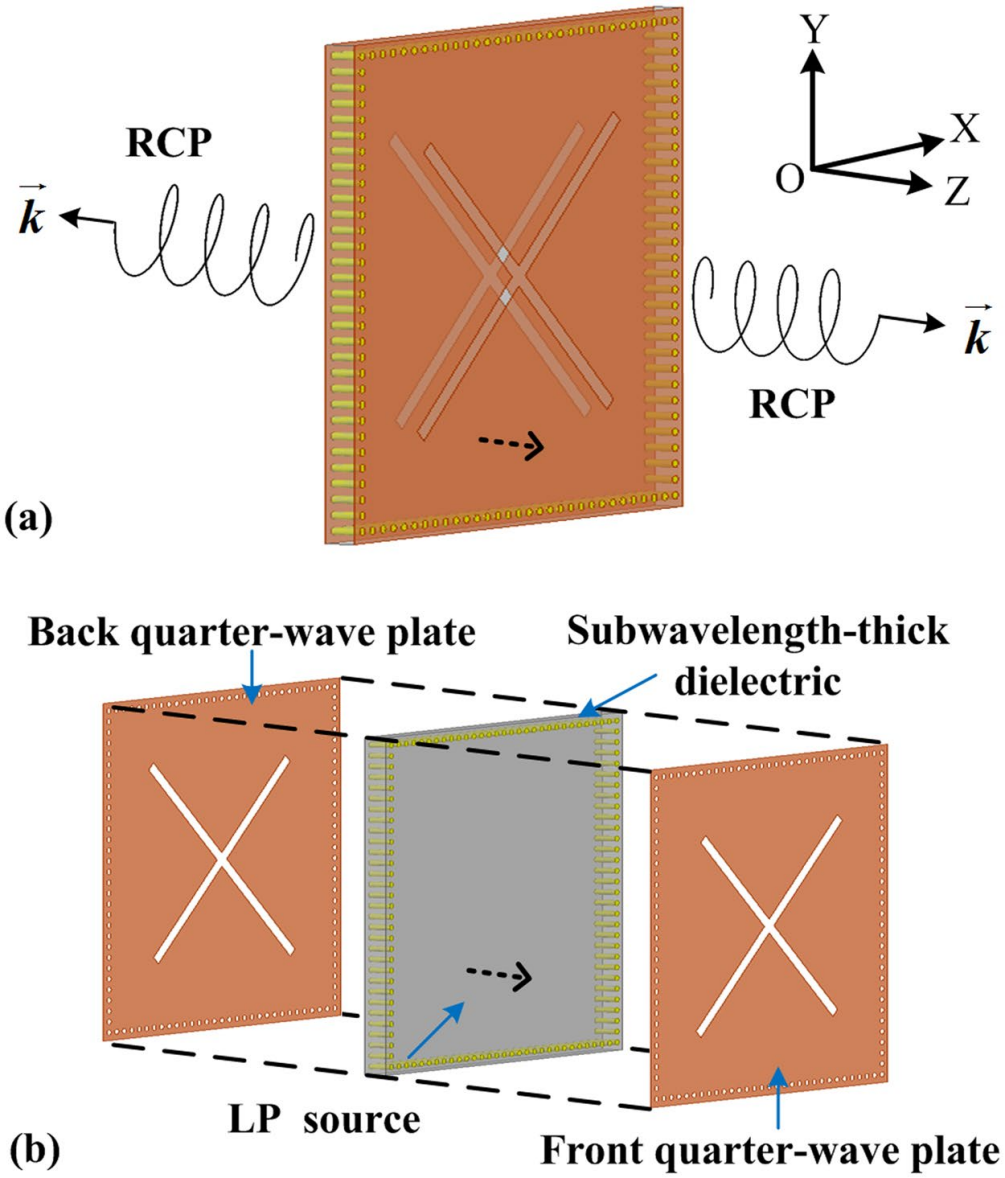
The general scheme of bidirectional CP wave generation with controllable helicity. (**a**) An example of RCP wave for +Z and −Z directions, (**b**) layered view of the ultrathin cavity structure, which consists of a pair of quarter-wave plates integrated with an ultrathin dielectric. A LP source is used to excite the ultrathin cavity.

The general scheme of bidirectional helicity-controllable CP wave generation is shown in Fig. 1(a). Without loss of generality, identical RCP wave for forward and back scattering or radiation in microwave are discussed as an example. As illustrated in Fig. 1(b), the proposed structure consists of a pair of back-to-back QWPs with crossed slots, separated by an ultrathin dielectric. A series of shorting wires are used to construct the metallic boundary condition as a cavity structure based on the low-cost PCB process^18^.

## Results

**Figure 2:**
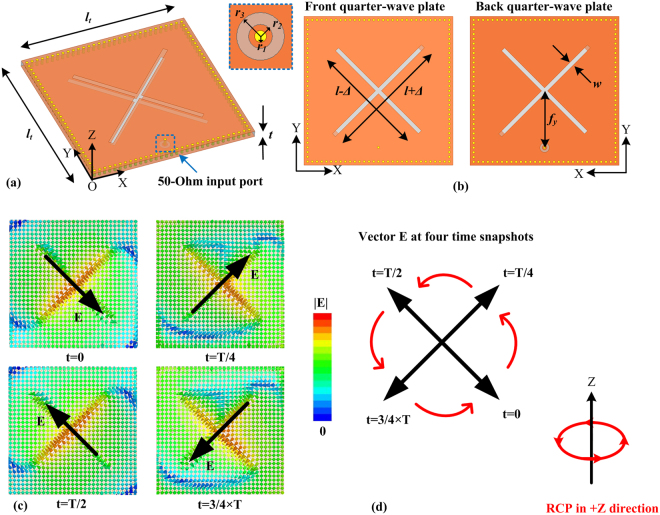
(**a**) Perspective view of the ultrathin cavity structure with dual quarter-wave plates with *l*_t_ = 0.478λ_0_ and *t* = 0.020λ_0_; the 50-Ohm input port is excited by a current source with the input power of 1 W; (**b**) detailed dimensions of the dual quarter-wave plates with an electric dipole source with *l* = 0.409λ_0_, *Δ* = 0.014λ_0_, *fy* = 0.191λ_0_, *w* = 0.014λ_0_, *r*_1_ = 0.004λ_0_, *r*_2_ = 0.008λ_0_, and *r*_3_ = 0.016λ_0_; field distributions of the ultrathin cavity structure in microwave with λ0 = 125.5 mm (*f*_0_ = 2.39 GHz) in a phase period: (**c**) vector E-field distributions in the XY-plane (1 mm above the front quarter-wave plate) at *f*_0_ in a period of T. (**d**) Illustration of the RCP in +Z direction based on the vector E distributions at four time snapshots in (**c**). At *f*_0_ = 2.39 GHz, *l*_t_ = 60 mm and *t* = 2.5 mm, *l* = 51.3 mm, *Δ* = 1.76 mm, *f*_y_ = 24 mm, *w* = 1.76 mm, *r*_1_ = 0.5 mm, *r*_2_ = 1.0 m, and *r*_3_ = 2.0 mm.

Based on the general scheme in Fig. 1, a practical ultrathin back-to-back QWPs cavity is constructed using the PCB process, as shown in Fig. 2(a). The thickness of the cavity is very small with a value of 0.02λ_0_ (λ_0_ is the operating wavelength in free space). A probe port is designed with the input impedance of 50 Ohm. As illustrated in Fig. 2(b), for the QWP design, the crossed slots with unequal length can introduce 90-degree phase difference between the orthogonal LP waves generated from the slots. The front QWP is with the longer slot along 45° and shorter slot along 135° in the XY-plane, generating RCP wave from LP incident field. To generate LCP wave, the longer slot should be positioned along 135° and shorter slot along 45°. The parameters ‘*l*’ and ‘*t*’ determine the operating frequency, and ‘*Δ*’ is used to optimize the CP performance, e.g., axial ratio (AR). The polarization state of the back QWP is controlled in the same rule. Figure 2(c) depicts the vector electric field distribution at the operating frequency, e.g., *f*_0_ = 2.39 GHz (λ_0_ = 125.5 mm) with the cavity thickness *t* = 2.51 mm. For t = 0 (i.e., 0° phase), the electric field is concentrated in the shorter slot with a half wavelength mode and polarized along −45° with respect to the horizontal axis. For t = T/4 (i.e., 90° phase), the half wavelength field distribution is along the longer slot with +45° polarization. For t = T/2 and t = 3/4 × T (i.e., 180° and 270° phases), the electric fields are with the +135° and +225° polarizations. The trace of electric field in Fig. 2(c) is summarized in Fig. 2(d). We can see a counterclockwise rotation trace at n × T/2 (n = 0, 1, 2, 3). In determining the type of circular polarization, we put thumb parallel to the propagating direction of the wave (+Z), and other four fingers parallel to the trace of vector electric field. As shown in Fig. 2(d), the polarization fulfills the right-hand rule, which means RCP along +Z direction.

The operating principle of the crossed-slot QWP is further described based on Jones calculus: the radiating wave is the product of the Jones matrix of the microwave element and the Jones vector of the incident wave (we assume the time convention exp(−iωt)).

1 $$(\begin{array}{c}{E}_{t}^{x}{e}^{-i(\phi +{{\rm{\Delta }}}_{x})}\\ {E}_{t}^{y}{e}^{-i(\phi -{{\rm{\Delta }}}_{y})}\end{array})=(\begin{array}{cc}{t}_{11} & {t}_{12}\\ {t}_{21} & {t}_{22}\end{array})(\begin{array}{c}{E}_{i}^{x}{e}^{-i\phi }\\ {E}_{i}^{y}{e}^{-i\phi }\end{array})=M(\begin{array}{c}{E}_{i}^{x}{e}^{-i\phi }\\ {E}_{i}^{y}{e}^{-i\phi }\end{array})$$

Here, ***M*** is the Jones matrices of the crossed-slot QWP, while ${E}_{i}^{x}{e}^{-i\phi }$, ${E}_{i}^{y}{e}^{-i\phi }$, ${E}_{t}^{x}{e}^{-i(\phi +{{\rm{\Delta }}}_{x})}$, and ${E}_{t}^{y}{e}^{-i(\phi -{\Delta }_{y})}$ are the incident x-polarized, incident y-polarized, transmitted x-polarized, and transmitted y-polarized electric fields, respectively. Due to the fact that the x-polarized wave is independent with the y-polarized wave, the parame*t*ers *t*_12_ and *t*_21_ should be zero. Thus, we obtain:

2 $${t}_{11}=\frac{{E}_{t}^{x}}{{E}_{i}^{x}}{e}^{-i{{\rm{\Delta }}}_{x}}$$

3 $${t}_{22}=\frac{{E}_{t}^{y}}{{E}_{i}^{y}}{e}^{i{{\rm{\Delta }}}_{y}}$$

Therefore, the Jones matrices of the RCP QWP can be expressed as:

4 $$M=(\begin{array}{cc}\frac{{E}_{t}^{x}}{{E}_{i}^{x}}{e}^{-i{{\rm{\Delta }}}_{x}} & 0\\ 0 & \frac{{E}_{t}^{y}}{{E}_{i}^{y}}{e}^{i{{\rm{\Delta }}}_{y}}\end{array})$$

For the incident LP wave,

5 $${E}_{i}^{x}={E}_{i}^{y}$$

For an ideal transmitted RCP wave,

6 $${E}_{t}^{x}={E}_{t}^{y},{{\rm{\Delta }}}_{x}-{{\rm{\Delta }}}_{y}=\pi /4$$

The physical meaning of metrics ***M*** is described as following. For a cavity-back slot radiator, at the resonant frequency *f*_0_, the phase curve crosses zero from capacitive (negative value) to inductive (positive value)^19^. When the length of slot is *l* + *Δ*, the operating frequency moves to *f*_0_ − *f*_*Δ*_; similarly, when the length of slot is *l-Δ*, the operating frequency moves to *f*_0_ + *f*_*Δ*_. It is worth to mention the values of *Δ* and *f*_*Δ*_ are quite small compared to *l* and *f*_0_, thus the magnitudes of the three cases are almost the same. Due to the detuning effect, at the resonant frequency *f*_0_, the wave from the longer slot is with an advanced phase of *Δ*_*x*_, and the magnitude is almost the same (i.e., ${E}_{t}^{x}={E}_{i}^{x}$). For the shorter slot, the wave is with a lag phase of *Δ*_*y*_, and the magnitude is also almost the same (i.e., ${E}_{t}^{y}={E}_{i}^{y}$). Therefore, by tuning the value of ‘*Δ*’, we may find ${{\rm{\Delta }}}_{x}-{{\rm{\Delta }}}_{y}=\pi /4$ at the operating frequency.

**Figure 3:**
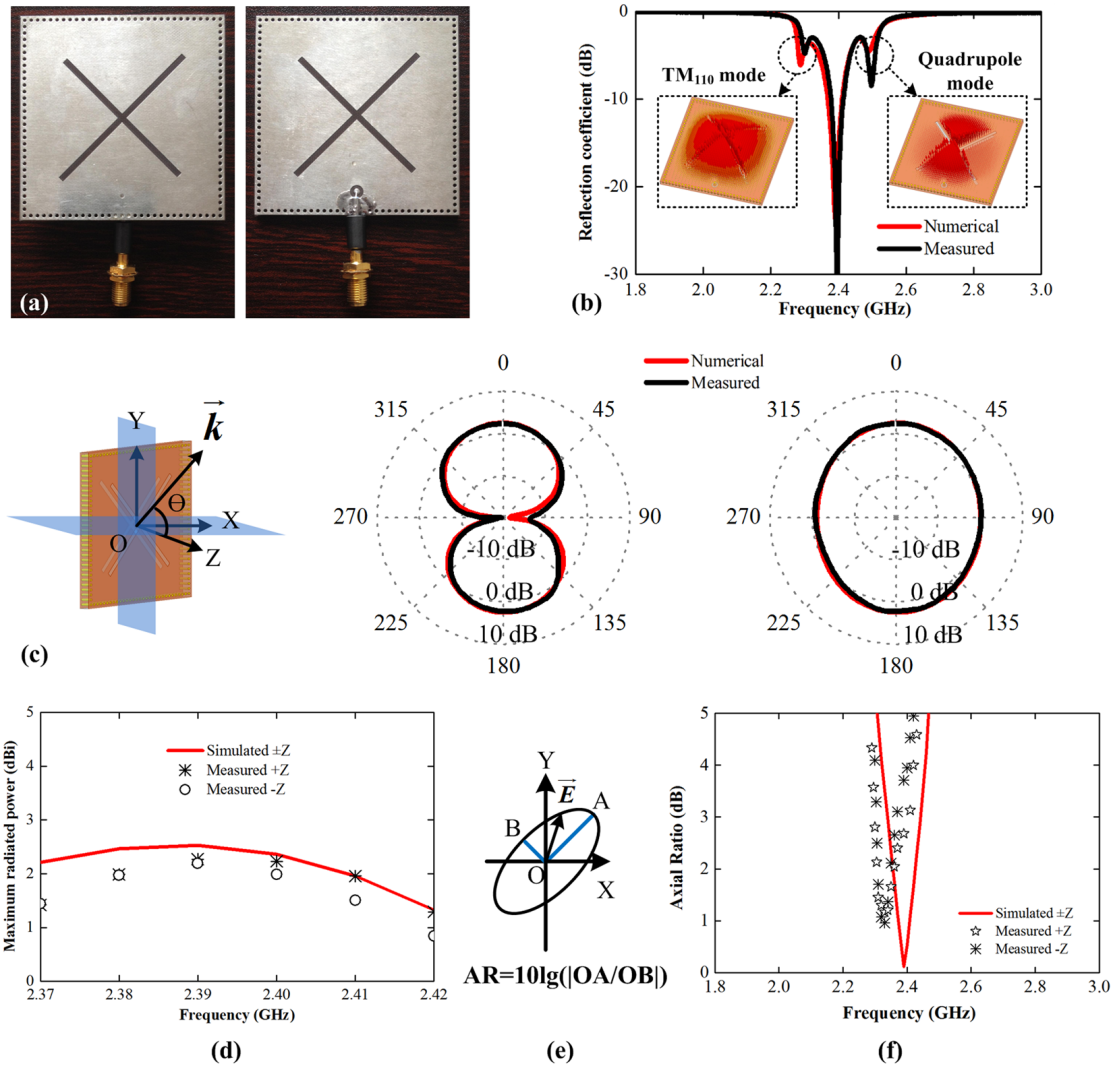
Experiment results of the ultrathin cavity structure in Fig. 2. (**a**) Front and back views of the fabricated prototype, a semi-rigid cable with SMA is used as the feeding. The inner conductor soldered on the one side and the outer conductor is soldered on the other side. (**b**) The measured and numerical magnitudes of reflection coefficients. (**c**) The measured and simulated angular patterns at 2.39 GHz in XZ-, and YZ- planes. (**d**) The measured and simulated maximum radiated power in the forward (+Z) and backward (−Z) directions. (**e**) Definition of axial ratio to express the quality of circular polarization, and (**f**) the measured and simulated axial ratio in the two directions.

To prove the design of ultrathin QWPs cavity, we have taken the numerical simulations and experimentally demonstrated the device. Without loss of generality, we simulated the cavity structure in microwave region of *f*_0_ = 2.39 GHz. The front and back views of the fabricated prototype are shown in Fig. 3(a). The magnitude of the reflection coefficient at the input port is given in Fig. 3(b). One can clearly see that the experimental spectrum (black curve) show three resonances at 2.28 GHz, 2.39 GHz, and 2.49 GHz, agreeing very well with the prediction form numerical results (red curve). As depicted in the insets: the lowest resonance is the fundamental TM110 mode of the cavity, and the highest resonance is the quadruple mode of the cavity. These two modes can’t provide CP wave and will not be used in the practical applications. The second resonance is the desired operating frequency: the simulated −10 dB impedance bandwidth is from 2.37 GHz to 2.41 GHz, centered at 2.39 GHz.

The angular profiles of RCP wave radiation are shown in Fig. 3(c), which are measured in a standard ETS far-field anechoic chamber using an E5071C vector network analyzer, agreeing well with the numerical results. It is clear seen that the bidirectional profile with the maximum radiated power towards to +Z and −Z directions. At *f*_0_ = 2.39 GHz, the maximum radiated power (normalized by incident power) is 2.2 dB higher than the ideal isotropic RCP radiation, indicating low dissipation in dielectric and port mismatch. The LCP wave is much smaller power than RCP wave, and too small to show. The measured and simulated maximum radiated powers in the ±z directions are given in Fig. 3(d). The measured maximum powers vary from 1.29 to 2.27 dBi in the +Z direction, and from 0.84 to 2.16 dBi in the −Z direction.

For the CP performance, we used definition of axial ratio (AR)^19^, also plotted in Fig. 3(e), to evaluate the states and quality of CP wave. The small value of AR means the excellent RCP performance for the radiated wave. Figure 3(f) illustrates the measured AR curves in the ±Z direction compared with the simulated results. The simulated AR curve in the ±Z directions are overlapped. The measured frequency ranges with AR < 3 dB are from 2.3 GHz to 2.41 GHz and from 2.31 GHz to 2.37 GHz for +Z and −Z directions, respectively. The difference between the measured ARs of the +Z and −Z directions attributes to the uncertain scattering from cables, which are used to connect the QWPs cavity and the equipment. It is worthy to mention again that the bidirectional CP waves with identical helicity are generated from an ultrathin cavity with only single dielectric layer.

## Discussions

**Figure 4:**
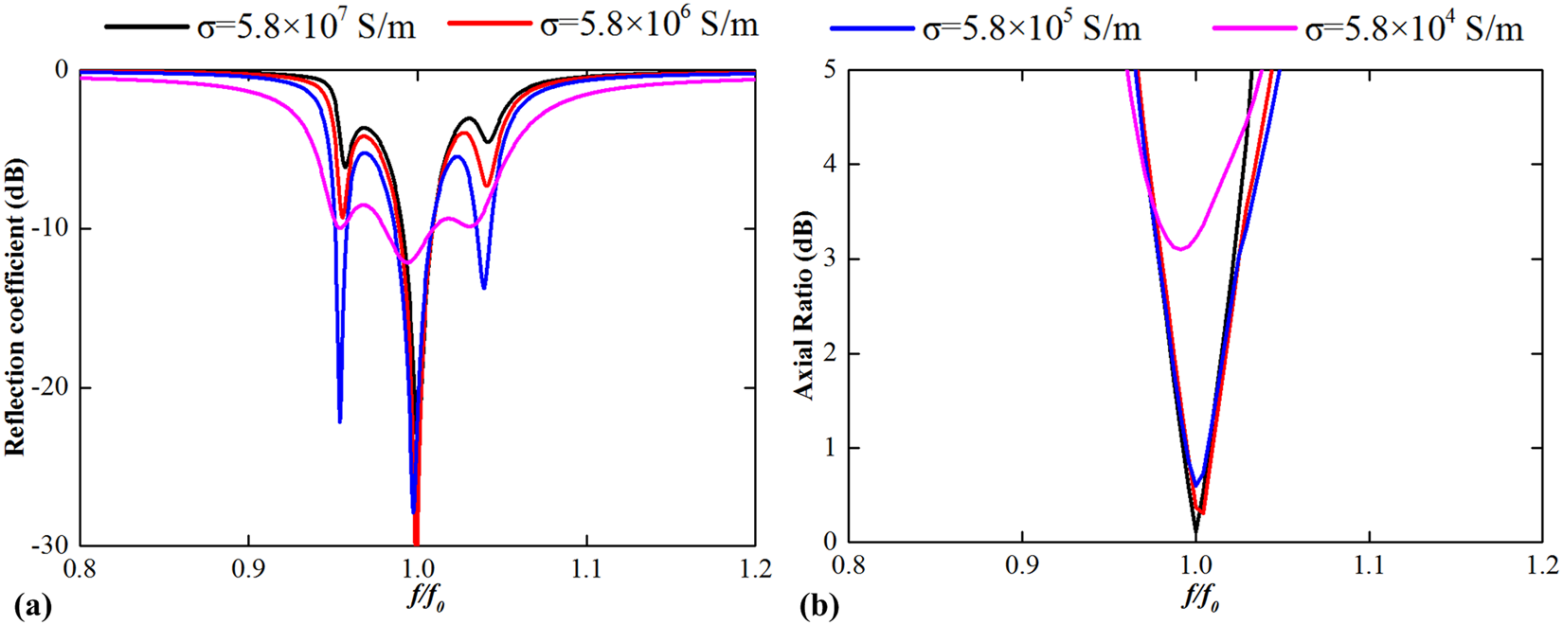
Parameter study of the ultrathin cavity structure of different electric conductivities. (**a**) Reflection coefficients and (**b**) axial ratios with different conductivities of copper as a function of normalized frequency.

The method of ultrathin back-to-back QWPs cavity can be translated from microwave to higher frequencies, if the conductivity can maintain at a high level. The electric conductivity of the bulk metal with a certain thickness slightly decreases as the operating frequency increases up to a certain range, e.g., THz region^20–23^. For this purpose, we simulated the AR performance of the same model in Fig. 2(a) with different electric conductivity values. As shown in Fig. 4, if the conductivity is higher than 5.8 × 10^5^ S/m, the AR performance almost remains unchanged, so do the radiation angular profile and maximum radiated power. The result reveals that the metallic boundary is significant for the CP state. When the conductivity decreases (i.e., metallic loss increases), the back-to-back QWPs are mutual coupled and the CP performance deteriorates. Thus, for higher frequency range (e.g. in THz region), in which the conductivity of metal decreases to a low value, illustrated as the pink curve in Fig. 4, the proposed device is no longer available. The undesired modes are significantly stronger compared to the operating mode, which is resonant at *f*_0_.

**Figure 5:**
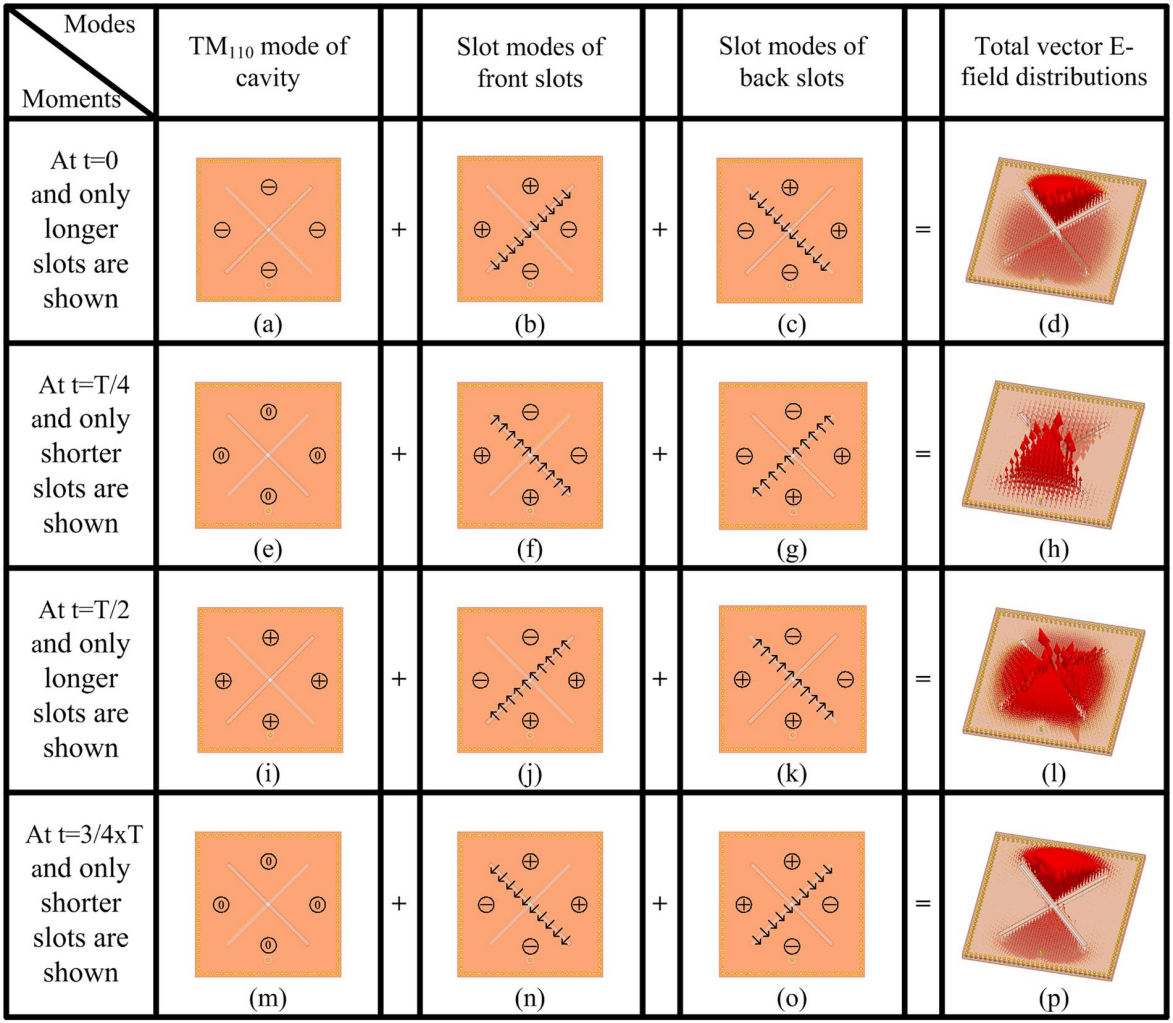
E-field analysis inside the cavity at 2.39 GHz within a period: modes schematics at t = 0 of (**a**) cavity, (**b**) front slot, and (**c**) back slot; (**d**) Simulated vector field snapshot by combining the field of (**a**–**c**); (**e**–**h**) fields at t = T/4; (**i**–**l**) fields at t = T/2; (**m**–**p**) fields at t = 3/4 × T. The black arrow lines denote the vector electric fields of the slots, the plus refers to the forward field, the minus refers to the backward field and zero refers to the zero-intensity field.

The detailed operating modes inside the cavity are analyzed and tabulate in Fig. 5. The black arrow lines denote the vector electric fields of the slots, and the symbols enclosed by circles denote the electric field directions: the plus refers to forward, the minus refers to backward and zero refers to the zero-intensity field. Figs (a), (e), (i), (m) plot the mode schematics of cavity within a period at 2.39 GHz. Similarly, Figs (b), (f), (j), (n) plot the mode schematics of the front slot and Figs (c), (g), (k), (o) plot the mode schematics of the back slot. The simulated vector field by combining the three modes are illustrated in Figs (d), (h), (l), (p). At 2.39 GHz, five kinds of the cavity modes exit: the fundamental TM110 mode, the front longer slot mode, the back longer slot mode, the front shorter slot mode, and the back shorter slot mode. The latter four modes are the dominant modes while the TM110 mode is the less dominant mode at 2.39 GHz, which means the TM110 mode is relatively weaker.

As to each slot mode, the resonant slot divides the square cavity into two triangular cavities and the electric fields inside the two cavities are phase reversed. The cavity is divided into four small triangular sectors by these slots. At t = 0 moment, the two longer slots are resonant while the two shorter slots are non-resonant, so three modes including the TM110 mode, the front longer slot mode, and the back longer slot mode exist, as shown in Row-2 respectively, where the non-resonant shorter slots are omitted. The dominant mode is the superposition of the front and back longer slot modes, which shows zero intensity in the two horizontal sectors and maximum intensity in the two vertical sectors with reversed phase. In addition to the weaker TM110 mode which shows backward field, the simulated total vector electric field distribution as shown in the last figure of the Row-2 is the combination of the three modes: it exhibits three in-phase sectors with one sector anti-phase. At t = T/4 moment, the two shorter slots are resonant while the two longer slots are non-resonant, so three modes including the TM110 mode, front shorter slot mode, and the back shorter slot mode exist, as shown in Row-3 respectively, where the non-resonant longer slots are omitted. The TM110 mode at t = T/4 moment has zero intensity. Thus, the total field is the superposition of the front and back shorter slot modes. As shown in the last figure of Row-3, the total electric field distribution shows zero intensity in the two horizontal sectors and maximum intensity in the two vertical sectors with reversed phase. The mode of the t = T/2 (3/4 × T) moment is the same with that of the t = 0 (T/4) moment except from the field phase is reversed.

The Bi-CP pattern with the same sense performance can be deduced from the front longer and shorter slot modes, and the back longer and shorter slot modes without considering the TM110 mode. In fact, the front longer and shorter slot modes are a couple of degenerated modes, so do the back longer and shorter slot modes. As seen in the fourth column, the front longer and shorter slots are operating alternatively every quarter period and they are orthogonal with each other, producing the RCP wave forward. In the same way, the backward RCP wave is generated as deduced from the sixth column.

## Conclusions

In conclusion, the bidirectional CP wave control is realized based on single-layer and ultrathin dielectric. A pair of back-to-back QWPs are integrated to form an ultrathin cavity with little interactions. The helicities of the bidirectional radiated CP waves can be set freely by properly arranging the layouts of the crossed slots of the front and back QWPs. As an example, we have designed and fabricated the back-to-back QWPs cavity to generate a bidirectional CP wave with the same helicity in the microwave domain, and can be adopted up to THz range, in which the conductivity of the metal has no obvious deterioration. The measured results show the potential applications in the circular polarization states control for bidirectional radiated wave.

## Methods

### Numerical simulation setup

The numerical results presented in this study were obtained using the commercial software of High Frequency Structure Simulator (HFSS, version 14) based on the finite element method (FEM). HFSS adopts adaptive mesh method with the mesh type of tetrahedron. The solution type was selected as ‘Driven Modal’, the excitation approach was selected as ‘Lumped Port’ with the port impedance of 50 Ohm and input power of 1 W which offers a current source connecting the probe and the metal on the back side, the maximum iteration number was set as 50, the standard convergence error was set as 0.01, the frequency sweep type was selected as ‘Fast’ and the radiation boundary was set to the six surfaces of a vacuum box which locates 0.4λ_0_ away from each dimension of the model. The dielectric material used in this study is Taconic TLX-8 with a relative permittivity of 2.55 and a loss tangent of 0.0019. The copper conductors on the front and back surfaces are with the thickness of 18 μm and with a typical conductivity of σ = 5.8 × 10^7^ S/m. The shorting wires were also made of the same kind of copper.

### Fabrication of QWPs cavity

The QWPs cavity is constructed using a standard PCB process (see Fig. 3(a)). The crossed slots are achieved by surface etching process, and the shorting wires around the cavity are achieved by via-hole punching process. As described in the main text, the material used is the Taconic TLX-8 with double copper layers (front and back). A semi-rigid cable with the type of SFT50-2 is utilized to excite the QWPs cavity. The inner conductor runs though the cavity from the back QWP and soldered on the front QWP, operating as the current source. The outer conductor is soldered directly on the back QWP. A SMA-K connector is used on the other end of the semi-rigid cable for measurement. The diameters of the inner and outer conductors are 0.51 mm and 2.2 mm, respectively. A series of magnetic beads were loaded outside the semi-rigid cable to prevent the radiation from the surface current on the outer conductor.

### Measurement setup for reflection coefficients

The reflection coefficient is measured using a N5071B vector network analyzer (VNA), with the frequency range is from 300 KHz to 9 GHz). The device under test (DUT) is connected to the VNA using a coaxial cable with the type of ST18-SMSM-1M. Before measurement, the VNA was calibrated using the Electronic Calibration Module 85093 C. The magnitude of reflection coefficient, e. g., the curves in Fig. 3(b), is calculated using the formula:

$$|R|=10\times \,{\rm{lg}}(\frac{{P}_{r}}{{P}_{i}})$$

here *P*_*i*_ is the input power injected into the DUT and *P*_*r*_ is the reflected power from the DUT to the VNA.

### Measurement setup of OTA measurement

The radiation patterns in Fig. 3(c), gain in Fig. 4(d), and AR in Fig. 4(f) are measured in a standard ETS far-field anechoic chamber with the dimension of 7.4 × 3.75 × 3.75 m^3^. During measurement, the DUT operates as the receiving antenna, and a dual-polarized horn with the model of 3164-05 (2-18 GHz) is used as the transmitting antenna. The DUT is fixed on a rotary table that is controlled by a multi-device controller with the model of 2090. The distance between the DUT to the stationary transmitting antenna is 5 m. The magnitude of the transmission is measured and recorded using an E5071C VNA (the frequency range is 300 KHz to 20 GHz). The radiation patterns in the two planes are obtained through rotating the DUT around its center in the XZ- and YZ- planes for a circle, respectively. The gains of the CP wave are obtained using the gain comparison method between the DUT and a standard receiving horn, whose gain is known and used for the calibration. The AR curves are obtained by comparing the maximum and minimum E-field intensities, as depicted in Fig. 3(e).

### Data availability

All data used to obtain our conclusions are present in the main paper. Additional data related to this paper may be requested from Y.L.
